# Review of Seneca Valley Virus: A Call for Increased Surveillance and Research

**DOI:** 10.3389/fmicb.2018.00940

**Published:** 2018-05-11

**Authors:** Xiangle Zhang, Zixiang Zhu, Fan Yang, Weijun Cao, Hong Tian, Keshan Zhang, Haixue Zheng, Xiangtao Liu

**Affiliations:** State Key Laboratory of Veterinary Etiological Biology, National Foot and Mouth Diseases Reference Laboratory, Key Laboratory of Animal Virology of Ministry of Agriculture, Lanzhou Veterinary Research Institute, Chinese Academy of Agricultural Sciences, Lanzhou, China

**Keywords:** Seneca Valley virus, infection, pathogenicity, immune response, diagnosis

## Abstract

Seneca Valley virus (SVV) has recently caused many vesicular diseases in pigs in different regions and countries. As a newly causative agent of porcine vesicular disease, SVV has evolved and spread quickly. It causes clinical signs similar to those of foot-and-mouth disease and results in significant economic losses. An increasing number of SVV outbreaks were reported in 2016 and 2017 in Brazil, United States, and China. However, few diagnostic methods have been established and no commercial vaccine has been available until now. Therefore, more attention needs to be paid to SVV, and urgent surveillance should be performed to prevent the spread of this virus. Although recent research has shed some light on SVV, there are still many aspects of the virus and the disease that are not yet fully understood, and many questions need to be resolved. This review presents current knowledge concerning SVV infection, epidemiology, pathogenicity, immune response, and diagnostic methods. This information will aid the design and adoption of effective prevention and control strategies to counter this viral pathogen.

## Introduction

Seneca Valley virus (SVV), also known as Senecavirus A, is a non-enveloped, positive-sense, single-stranded RNA virus that belongs to the genus *Senecavirus*, family *Picornaviridae* (Knowles and Hallenbeck, 2005; Hales et al., 2008). SVV was first identified incidentally as a contaminant in cell culture medium during cultivation of PER.C6 cells (transformed fetal retinoblast cells) (Reddy et al., 2007). SVV does not affect humans and it is not pathogenic to normal human cells (Koppers-Lalic and Hoeben, 2011); however, it is an oncolytic virus that can propagate in human tumor cells (Reddy et al., 2007; Wadhwa et al., 2007). Therefore, after the first isolation of SVV, it has been used as an oncolytic virotherapy candidate in humans (Reddy et al., 2007; Rudin et al., 2009). The first isolated SVV (SVV-001) shows a strong cytotoxic effect on small-cell lung cancer cell lines and solid pediatric cancer cell lines (Reddy et al., 2007). A murine model of metastatic retinoblastoma was used to evaluate the potential therapeutic role of SVV-001, which is effective in suppressing invasive and metastatic retinoblastoma with no adverse events (Wadhwa et al., 2007). A first-in-human, first-in-class Phase I clinical evaluation of SVV in patients with small cell lung cancer with neuroendocrine features was also conducted, which indicated that SVV-001 was safe and had antitumor activity in patients (Rudin et al., 2011). A Phase II study of SVV-001 in patients with small-cell lung cancer is ongoing. However, how to minimize viral clearance rate and development of neutralizing antibodies is still a problem that hinders the development of clinical trials (Burke, 2016).

Six picorna-like viruses were isolated from pigs in the United States between 1988 and 2005, which showed a variety of clinical symptoms. They were closely related to each other and to SVV by sequencing analysis. Furthermore, each of these viruses is serologically related to the others, as well as to SVV-001 (Knowles and Hallenbeck, 2005). This implies that SVV might have existed for a long time in pigs in the United States. In 2007, 25–30% of 187 pigs from a Canadian market had broken vesicles along the coronary band, and among them approximately 80% of the pigs were lame. The clinical vesicular lesions were indistinguishable from those of vesicular diseases caused by foot-and-mouth disease virus (FMDV), swine vesicular disease virus (SVDV), vesicular stomatitis virus (VSV), or vesicular exanthema of swine virus (VESV). No positive results were identified by the polymerase chain reaction (PCR) tests for these viruses. However, SVV was identified as positive and was proposed as a causative agent of the presented disease (Pasma et al., 2008). After 2014, a sudden increase in outbreaks of vesicular diseases in pigs appeared in different countries (especially in Brazil, United States, and China) and SVV was determined to be the etiological agent (Joshi et al., 2016a,b; Leme et al., 2016a; Saeng-Chuto et al., 2017; Sun et al., 2017; Zhang et al., 2017; Zhu et al., 2017). Therefore, detection of SVV infection, development of diagnostic tools, improvement of surveillance and treatment and provision of effective control measures are essential to mitigate potentially negative impacts of this emerging infectious agent.

## Epidemiology

Seneca Valley virus has only one serotype, and is the only single species in the genus *Senecavirus*. Swine are thought to be a natural host of SVV and previous studies have indicated that SVV is not prevalent in humans. Serological surveys have revealed the presence of specific SVV antibodies in pigs, cattle, and mice, but not in humans (Pasma et al., 2008; Koppers-Lalic and Hoeben, 2011). SVV has been detected and isolated from vesicular lesions and tissues of affected pigs, mouse feces, mouse small intestine, and environmental samples. In addition, SVV RNA has been detected in houseflies collected from affected pig farms and from a farm with no history of vesicular disease (Joshi et al., 2016b). These findings suggest that pests (mouse and houseflies) might play a role in the epidemiology of SVV, however, no direct evidences has confirmed the transmission route. Neutralizing antibodies to SVV have been detected in swine, cattle, and mice (Knowles and Hallenbeck, 2005; Joshi et al., 2016b). Whether cattle, wild cloven-hoofed animals or mice are directly infected by SVV or not, is still unknown. FMDV, another picornavirus that also affects pigs and causes similar vesicular disease, spreads by direct contact or exposure to aerosolized virus, and it also affects various wild cloven-hoofed animals (Rweyemamu et al., 2008; Stenfeldt et al., 2016). However, many aspects of SVV infection characteristics, host range, and epidemiology remain unknown.

Cases of vesicular diseases of unknown etiology have been reported in pigs in New Zealand (Montgomery et al., 1987) and Australia in the 1980s (Munday and Ryan, 1982), the United Kingdom in 2007 (International Society for Infectious Diseases, 2015), and Italy in 2010 (Sensi et al., 2010). The diagnostic results for FMDV, SVDV, VSV, and VESV were negative; but there was no diagnosis for SVV. Whether SVV also exists in these countries should be further investigated. The first SVV-positive case in pigs was reported in Manitoba, Canada in 2007, which reminds us of the potential association of SVV with idiopathic vesicular disease (Pasma et al., 2008). The case that led to SVV being considered as an etiological agent of vesicular disease occurred in 2010 in the United States, and the lesions caused by SVV were first described (Singh et al., 2012). Before 2014, SVV was only identified in the United States and Canada, and it is thought that SVV might have been silently circulating throughout the United States for several years, as far back as 1988. Because six picorna-like viruses, isolated from pigs showing a variety of clinical symptoms in the United States between 1988 and 2005, have been shown to be closely related to each other and to SVV by sequencing analysis, and these viruses have been shown to be serologically related to each other, as well as to SVV-001 (the first identified SVV strain) (Knowles et al., 2006).

At the end of 2014, an outbreak of SVV infection in pigs was first reported outside of the United States and Canada in Brazil (Vannucci et al., 2015). A 10-year (2007–2016) retrospective serological survey for SVV in Brazil has provided robust evidence that SVV was not present in the major Brazilian pig-producing regions before 2014 (Saporiti et al., 2017). It is suggested that SVV was imported into Brazil in 2014. Since 2015, SVV infection began to spread rapidly among pigs in different age groups in more regions and countries. Moreover, 2015 is considered as a turning point for enlarged spread of SVV. Outbreaks of SVV infection in pigs of different ages were reported in at least seven regions in Brazil in 2015 (Leme et al., 2015, 2016a, 2017). SVV infections reported in the United States and Canada were only observed in adult pigs that cause vesicular diseases; however, for the first time, the clinical manifestations in piglets were reported in Brazil, and the morbidity in neonatal pigs were higher than that in the adult pigs. Meanwhile SVV infection can lead to acute death of neonatal piglets (Leme et al., 2015, 2016a, 2017). This apparently indicates that there is an evolution of SVV into a more virulent phenotype. In the same year, two cases were reported in Ontario and Manitoba, Canada. The complete coding sequences of SVV from the two clinical cases and nine assembly yard environmental samples were analyzed. A rapid genetic variations accumulation driven by a high nucleotide substitution rate and purifying selection was observed for these isolates, it suggested that these SVV strains had been evolving constantly (Xu et al., 2017). It was also reported for the first time in Guangdong Province, China in 2015, that both adult and newborn pigs were infected in this outbreak (Wu et al., 2016). From 2015 to 2016, SVV infection in pigs was reported in more regions in Brazil, the United States, China, Canada, Colombia, and Thailand with an extensive distribution (Rademacher et al., 2015; Joshi et al., 2016a,b; Leme et al., 2016a; Saeng-Chuto et al., 2017; Sun et al., 2017; Wang et al., 2017). New cases of SVV were reported in the United States and China in 2017 (Sturos, 2017; Zhang et al., 2017; Zhu et al., 2017; Liu et al., 2018), and the 2016 is considered as a turning point for the SVV epidemiology in China. Two different subclades have been identified. Viruses that caused outbreaks before 2016, shared higher nucleotides identities with the strains which were isolated in Canada and Brazil. However, all the SVV strains which were reported after 2016 were more closely related to the United States strains (Zhang et al., 2017; Zhu et al., 2017). A certain degree of genetic distance has been determined for the strains in 2017 comparing with previous strains, suggesting the constant and rapid evolvement of SVV (Zhang et al., 2017). According to recent studies, all of currently reported SVV cases in 2017 in China were identified in adult pigs including finishing pigs and sows; however, piglets did not show any clinical manifestations of disease. The recent Chinese SVV strains are closely related to current United States strains. The previous Chinese strains (reported in 2015) that can cause acute death in newborn pigs are closely related to the previous Brazilian strains. Whether different strains from different regions reveal different virulent phenotypes or not should be investigated. More surveillance and investigation on the incidence of vesicular diseases in pigs should be performed to understand the current spread and evolution of SVV in different regions and countries.

## Infection, Illness, and Pathogenesis

**FIGURE 1:**
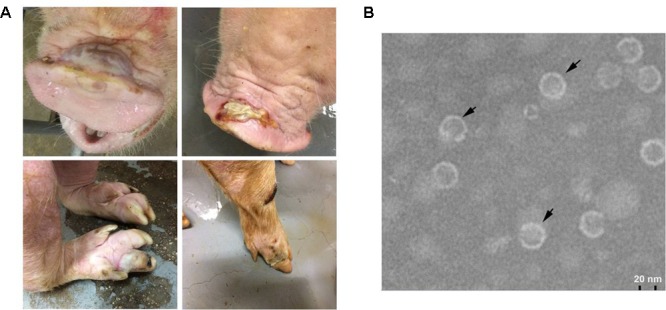
Lesions caused by SVV infection in pigs, and virions recorded by electron microscopy. **(A)** Fluid-filled vesicles on the snouts (upper panel), and ulcerative lesions on the foot (lower panel). **(B)** SVV virions observed using electron microscopy.

Seneca Valley virus infection causes vesicular disease that is indistinguishable from the clinical signs of FMD, swine vesicular disease, vesicular stomatitis, or vesicular exanthema (Leme et al., 2015). The clinical signs in SVV-infected pigs include fluid-filled and ruptured vesicles or ulcerative lesions on the snouts and coronary bands, lameness, anorexia, lethargy, cutaneous hyperemia, and fever (**Figure 1A**) (Leme et al., 2015; Zhu et al., 2017). The affected adult pigs often present with difficulty in moving, decreased feed intake, and daily weight gain. The viremia period lasts approximately 7 days, and the clinical signs may persist for 2–14 days (Maggioli et al., 2017). According to an experimental study, the finishing pigs (*n* = 8) challenged by an SVV strain (5 × 10^8.07^ TCID_50_) isolated in the United States in 2015 presented lameness and lethargy by day 4 post-infection (pi), and these manifestations persisted for 2–10 days. The vesicular lesions were found either on feet or snout and in some cases these lesions were found both on feet and snout (6/8 pigs). Viral RNAemia was detected in serum during a period of approximately 7 days (3–10 days pi). The presence of SVV in tissues was assessed following convalescence from disease. SVV was detected in the lungs, lymph nodes, liver, spleen, small and large intestines, and tonsils of infected finishing pigs between 3 and 5 days post-infection, and it showed that SVV has a tropism for lymphoid tissues, with a higher level in the tonsils. Therefore, the tonsils are likely to be considered as the primary replication site for SVV that can be selected for virus isolation. The virus shedding lasts up to 28 days and can be detected in oral and nasal secretions and also in feces. The highest viral peak occurs between 1 and 5 days post-infection in oral secretions, suggesting the acute nature of the vesicular disease caused by SVV (Joshi et al., 2016a). Another infection study using a Chinese SVV strain isolated in 2017 was performed in finishing pigs. The finishing pigs were challenged by the virus at a dose of 3 × 10^9^ TCID_50_. All these pigs showed fluid-filled vesicles on the snout and ulcerative lesions on the coronary band. Moreover, among them, several pigs developed fever at 2–6 days post-challenge (dpc) which lasted for 1–3 days. The viral RNA can be detected in the blood after 1–7 dpc and the RNAemia disappeared after 9 dpc (Yang et al., 2018). These studies suggest SVV induces acute and self-limiting vesicular disease in adult pigs.

Seneca Valley virus infection has also been associated with pig neonatal mortality [recently named epidemic transient neonatal losses (ETNLs)], especially in pig herds with SVV-affected sows (Canning et al., 2016; Gimenez-Lirola et al., 2016; Leme et al., 2016b). A longitudinal field study in a swine breeding herd in the United States in 2015 presented that SVV infection was associated with neonatal morbidity. Neonatal morbidity and mortality mainly occurred in piglets aged ≤7 days and SVV RNA was detected from multiple tissues, blood, and excretions (Gimenez-Lirola et al., 2016). The pathological, immunohistochemical, and molecular evaluation of 12 piglets aged ≤5 days with cutaneous, enteric, and neurological disorders in Brazil in 2015 showed that multiple tissues of these piglets were SVV antigen and RNA positive. The positive results for the SVV RNA and antigen detection in 1- to 2-day-old piglets suggested that vertical transmission might be a form of dissemination (Leme et al., 2016b). Another study of clinical manifestations of SVV infection in 10 piglets in Brazil in 2015 also suggested that there was an association of SVV infection with neonatal morbidity. Petechial hemorrhages of the kidneys and ulcerative lesions of the tongue and coronary bands were the most frequent gross manifestations in these piglets (Leme et al., 2016a). Interstitial pneumonia was observed as the predominant histopathological alteration in SVV-infected piglets. SVV antigen could be detected in the uroepithelium of many infected piglets, which implies that urine might be a possible route for the dissemination of SVV (Leme et al., 2016a). Oliveira et al. (2017) further investigated the possible SVV-induced lesions by examining spontaneous infections in newborn piglets. 54 piglets with clinical signs of ETNL were included the study, and 80% of these pigs (43/54) were SVV antigen and RNA positive. 35/43 (81%) of these SVV positive piglets died due to ETNL between 2 and 5 days of age. This suggests the clinical sings of SVV are more intense in new-born piglets that leads to sudden death of the piglets. Ballooning degeneration of the transitional epithelium was determined as the most frequent histopathological lesion associated with SVV in these newborn piglets. The urinary bladder, choroid plexus, renal pelvis, oral mucosa, and ulcerative lesions presented more histopathological alterations and showed immunoreactivity to SVV. These tissues were recommended for histopathological and immunohistochemical diagnosis of SVV in newborn piglets (Oliveira et al., 2017). Resende et al. (2017) found that SVV-infected litters of piglets showed transient sudden death, and SVV RNA was detected in the brain, spleen, lungs, liver, colon, small intestine, nasal sinus, tongue, and tonsils using an RNA-based *in situ* hybridization. Dall Agnol et al. (2017) detected SVV from different tissues of neonatal piglets using a TaqMan-based quantitative reverse transcription PCR (qRT-PCR) assay and suggested that the lungs, heart, urinary bladder, kidneys, spleen, tonsils, and small intestine could be selected for SVV detection in piglets. All these studies confirm the participation of SVV in the multiple lesions in piglets, and the piglets infected by SVV develop multisystemic diseases. However, absence of lesions in ETNL cases has also been reported in Brazil and in the United States (Vannucci et al., 2015; Canning et al., 2016; Segalés et al., 2016). In addition, most of SVV cases in China were reported in sows and finishing pigs. Therefore, the hypothesis of SVV being a primary causative agent for acute neonatal mortality remains to be elucidated. Although significant progress has been made and understanding of SVV infection and disease has been significantly improved, there is still much to be learned about the virus and its associated disease.

Receptors for SVV in pig cells remain unknown. However, it is reported that the carbohydrate sialic acid is a component of the SVV receptor in human pediatric glioma. Removal or blocking of sialic acid modestly reduces SVV infection (Liu et al., 2013). Sialic acids are used by many viruses as receptors and are involved in host range restrictions, tissue tropism and pathogenesis (Alexander and Dimock, 2002). Heparan sulfate is a carbohydrate that has been identified as the cellular receptor for FMDV (O’Donnell et al., 2008). Whether heparan sulfate is involved in the binding of SVV to pig cells should be investigated. Recently, human anthrax toxin receptor 1 (ANTXR1) has been identified as a cellular receptor for SVV in human tumor cells using genome-wide loss-of-function screening (Miles et al., 2017). ANTXR1, a single-pass transmembrane glycoprotein, shares common features with the immunoglobulin superfamily proteins which include many picornavirus receptors, and is also a tumor endothelial marker in humans (Carson-Walter et al., 2001). Knockout of ANTXR1 significantly leads to the loss of SVV permissivity. ANTXR1 is necessary for permissivity *in vitro* and *in vivo*. SVV capsid interacts directly and specifically with ANTXR1. This interaction is required for SVV binding to the host cells. ANTXR1 is frequently expressed on the surface of tumor cells compared with normal human cells (Miles et al., 2017). This may explain that why SVV does not infect normal human cells. However, SVV infects normal swine cells. Swine ANTXR1 has been predicted by automated computational analysis (GenBank Accession No. XM_003125066). It should be further investigated that whether ANTHRAX1 is also a cellular receptor in swine cells or not.

## Virology and Immunology

**FIGURE 2:**
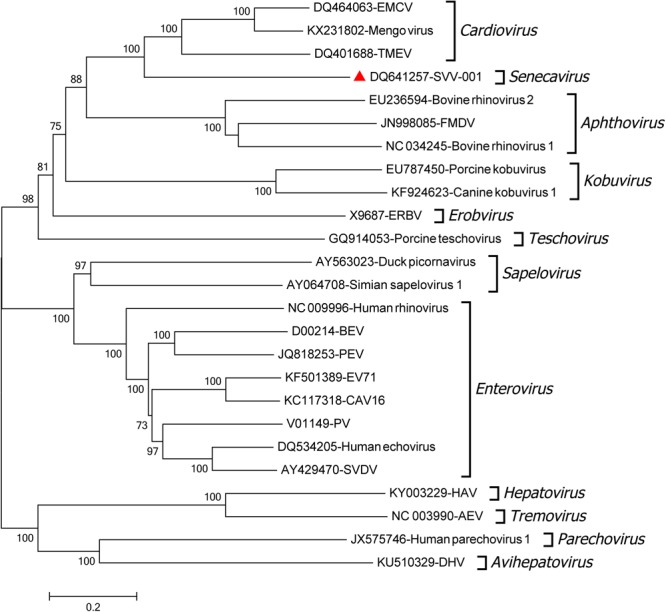
Phylogenetic comparison of the complete genome of SVV with those of other picornaviruses. Midpoint-rooted Neighbor-joining tree shows the relationships between the viral genomes of different picornaviruses. The tree was produced in MEGA 6.06 using the Kimura 2 parameter nucleotide substitution model. The numbers of bootstrap replicates were set as 1000.

The first SVV genome was determined in 2007 (SVV-001 strain) which has typical genomic feature of other picornaviruses including the standard L-4-3-4 layout (Hales et al., 2008). The virus genome is 7 200-7 300 bp in length, with 666 or 667 nucleotides in the 5′ untranslated region (UTR) and about 70 nucleotides in the 3′ UTR. The viral genome includes a single large open reading frame encoding a polyprotein (Hales et al., 2008; Xu et al., 2017). The polyprotein undergoes a series of proteolytic processes by viral proteinase to produce the mature viral proteins (12 polypeptides) including leader protein (L^pro^), VP4, VP2, VP3, VP1, 2A, 2B, 2C, 3A, 3B, 3C^pro^ and 3D^pol^, and the four structural proteins make up the round viral particles with a diameter of ∼30 nm (**Figure 1B**) (Hales et al., 2008; Yang et al., 2018). The 5′ UTR of SVV contains a type IV internal ribosome entry site element that is closely related to that of porcine teschovirus-1 but distinct from that of other picornaviruses (Hales et al., 2008; Belsham, 2009). Phylogenetic comparison of the complete genome of the first identified SVV (SVV-001) with those of other picornaviruses suggests that SVV is related more closely to members of the genus *Cardiovirus* (including encephalomyocarditis virus) and *Aphthovirus* (including FMDV) (**Figure 2**).

Seneca Valley virus infection develops a rapid neutralizing antibody (NA) response in sows and finishing pigs regardless of the clinical manifestations of the disease (Gimenez-Lirola et al., 2016; Joshi et al., 2016a). Mice also develop NAs following intravenous injection of SVV (Reddy et al., 2007). High levels of NAs can be detected at about 5 days post-infection, and the peak NA titers occur at about 7 days post-infection (Yang et al., 2012; Maggioli et al., 2017). The early NAs at the fifth day might be predominantly composed of IgM antibodies. SVV-specific IgG antibodies appear later and are first detected in the serum on day 7 post-infection. VP2 and VP3 mainly contribute to the IgM and early NA response after SVV infection. The IgM concentration peaks between days 7 and 10 post-infection and shows a rapid decrease after 14 days post-infection which becomes undetectable by 21–35 days (Yang et al., 2012; Maggioli et al., 2017). Similar to IgM responses, SVV-specific IgG antibodies are also primarily against VP2 and VP3. VP1 and VP3 IgG antibodies decline following resolution of the disease, and the antibodies last for ∼1 month, and are undetectable at 35 days post-infection. However, VP2 specific IgG antibodies can be detected until day 35 post-infection which might be responsible for the later neutralizing activity after 35 days post-infection (Yang et al., 2012; Joshi et al., 2016a; Maggioli et al., 2017). SVV infection also induces a marked T cell response that is characterized by an increased frequency of αβ T cells, especially CD4^+^ T cells that are initially detected by day 7 post-infection and increased in frequency until day 14 post-infection. An increase of CD8^+^ and double-positive CD4^+^CD8^+^ T cells associated with interferon-γ production are observed after day 10 post-infection (Maggioli et al., 2017).

The innate immune system plays a significant role in recognition of viral components and triggering the antiviral response (Thompson et al., 2011). Many viruses have evolved subtly and equipped themselves to block and evade host antiviral responses (Kawai and Akira, 2006). Pattern-recognition receptor (PRR)-initiated antiviral responses are particularly important in the innate immune responses. SVV, as an RNA virus, is mainly recognized by retinoic acid-inducible gene I (RIG-I)-like receptors (RLRs). However, SVV infection does not trigger an early host innate immune response and type I interferon production in human embryonic kidney 293T cells. 3C protein induces the cleavage of the adaptor molecules of type I interferon pathway MAVS, TRIF, and TANK through its protease activity. This effect blocks activation of the RLR pathway and inhibits type I interferon production (Qian et al., 2017). In addition to regulation of RLR pathway activation, incubation with apoptosis inhibitors significantly restrict virus replication in 293T cells, suggesting that SVV infection induces host cellular apoptosis to facilitate viral replication (Qian et al., 2017). These studies indicate that SVV disrupts host defense system by various different mechanisms in virus-infected cells.

## Diagnosis and Prevention

Diagnosis of SVV is crucial for SVV control and prevention. Diagnosis of SVV could be performed by detection of virus, viral antigens, or viral RNA. Many types of cells have been used for isolation of SVV, including human cancer, swine, and engineering cell lines. These include human retinoblast cells PER.C6 (Reddy et al., 2007), human lung cancer cell monolayers NCI-H1299 (Yang et al., 2012), human non-small-cell lung carcinoma cells H1299 (Joshi et al., 2016a), human embryonic kidney cells HEK293T (Qian et al., 2017), swine testis cells SK-RST (Segalés et al., 2016), the porcine kidney cells PK-15 and IBRS2 (Zhu et al., 2017), and baby hamster kidney cells BHK21 (Qian et al., 2016). SVV infection causes cytopathic effects in these cells at different titers. Plaque morphology can be observed in BHK-21 cells after SVV infection (Qian et al., 2016). A virus neutralization test (VNT) assay is available for assessing antibodies against SVV in infected cells. Our recent data show that SVV particles are round with a diameter of ∼30 nm (**Figure 1B**) (Yang et al., 2018). Observation of viral particles by electron microscopy is not diagnostic, but can aid diagnosis of SVV infection. Electron microscopy is a tool that enables fast visualization of viruses and their morphological identification. Additionally, the electron microscopy may provide additional findings that can contribute to the understanding of viral pathogenesis. Identification of SVV by immunofluorescence assay using anti-SVV VP1 protein antibody has been reported as a method for detection of SVV in infected cells (Qian et al., 2016).

Immunohistochemical (IHC) staining and *in situ* hybridization are also established methods to identify SVV antigen and RNA in tissue samples (Joshi et al., 2016a; Leme et al., 2016a; Resende et al., 2017). SVV RNA can be detected from the ruptured snout, necrotizing lesions in the tongue and skin of the coronary band of infected adult pigs (Resende et al., 2017; Zhu et al., 2017; Liu et al., 2018), and pulmonary, myocardial and tonsilar tissues are the most important biological samples in which the virus can be detected in piglets (Leme et al., 2016b; Dall Agnol et al., 2017). A specific SVV monoclonal antibody was developed by Yang et al. (2012) for serodiagnosis, and a competitive enzyme-linked immunosorbent assay (cELISA) was established using this antibody (Yang et al., 2012). This method was validated in 2017 by detecting SVV antibodies in serial bleeds from SVV outbreaks. The results indicate a strong agreement of the test results among cELISA, VNT and an immunofluorescent antibody test, suggesting that these tests are suitable for serological detection of SVV in pigs (Goolia et al., 2017). An indirect ELISA for SVV VP2 antibody detection was further established recently that shows high sensitivity and specificity (Dvorak et al., 2017). For SVV RNA detection, RT-PCR is the most commonly used method. A series of conventional RT-PCR and qRT-PCR methods targeting different genes of SVV have been developed (Knowles et al., 2006; Leme et al., 2015; Bracht et al., 2016; Wang et al., 2016; Fowler et al., 2017). These RT-PCR methods could be used for rapid detection of SVV in vesicular diseases, and many isolates have been determined using these methods. Combined use of the different methods will further improve the accuracy of SVV diagnosis.

As no SVV commercial vaccines have been developed currently and immunization with FMDV vaccine shows no cross protection against SVV infection (Zhu et al., 2017), control of SVV infection in pigs has to depend on improvement of the management, feeding conditions, and environment of pig farms. Enlarged surveillance on the incidence of SVV infection and disinfection of the feeding facilities and the environment are essential to reduce the risk of occurrence. Recently, our laboratory has developed an inactivated SVV vaccine that can elicit NAs and protect pigs against SVV infection (Yang et al., 2018). It is a vaccine candidate that can be potentially used for limiting the transmission of SVV. Besides, development of a bivalent vaccine consisting of FMDV and SVV probably will be beneficial for controlling of porcine vesicular diseases.

## Future Perspectives

In recent years, SVV has demonstrated its capacity to evolve and cause morbidity and mortality in pigs, accompanying the expansion into new countries. Surveillance, epidemiological investigations and genetic characterization of SVV associated with vesicular diseases and neonatal mortality in pigs are important for controlling SVV and supporting the development of specific and effective diagnostic tests. The lack of available commercial vaccines, and because SVV is not included in the OIE Terrestrial Animal Health Code, means that the increasing incidence of SVV infection in pigs in different countries will continue to cause unpredictable and substantial outbreaks. Therefore, in response to this threat, animal administration authorities and farm veterinarians must show continued awareness of this disease and remain vigilant in their surveillance, treatment, and provision of effective control measures. Further evaluation and study of the pathogenesis of SVV in swine, transmission among swine, and development of effective commercial vaccines will help limit the spread of SVV.

## Author Contributions

XZ, HZ, and XL conceived the study and wrote the manuscript. ZZ, FY, WC, HT, and KZ collected data, drew the figures, and compiled the tables.

## Conflict of Interest Statement

The authors declare that the research was conducted in the absence of any commercial or financial relationships that could be construed as a potential conflict of interest.
